# The ability of the prognostic nutritional index to predict short-term mortality in geriatric acute heart failure

**DOI:** 10.1186/s43044-024-00604-0

**Published:** 2025-01-06

**Authors:** Hilal Akça, Hatice Şeyma Akça, Abuzer Özkan, Serdar Özdemir

**Affiliations:** 1 Department of Anesthesia and Reanimation, Başakşehir Çam Ve Sakura City Hospital, İstanbul, Turkey; 2 Department of Emergency Medicine, Karaman Education and Research Hospital, University of Karamanoğlu Mehmet Bey, Karaman, Turkey; 3 Department of Emergency Medicine, Bağcılar Education and Research Hospital, University of Health Sciences, İstanbul, Turkey; 4 Department of Emergency Medicine, Ümraniye Education and Research Hospital, University of Health Sciences, Site Mahallesi, Adıvar Sokak, No 44/15, Ümraniye, İstanbul Turkey

**Keywords:** Prognostic nutritional index, Acute heart failure, Albumin, Lymphocyte

## Abstract

**Background:**

Heart failure is a critical cardiovascular condition, necessitating comprehensive treatment approaches and contributing to elevated mortality rates. This study aimed to evaluate the effect of the prognostic nutritional index (PNI) on the prognosis of geriatric patients diagnosed with acute heart failure.

**Results:**

A total of 104 patients were included and evaluated retrospectively in this study; 57.7% of them were females, and 19.24% of the patients died. A statistically significant difference was identified between high (≥ 35.6) and low PNI (< 35.6) groups in terms of lymphocyte count, neutrophil–lymphocyte ratio, C-reactive protein, and albumin (*p* values: < 0.001, < 0.001, 0.011, and < 0.001, respectively). The area under the curve (AUC) value for albumin was 0.53 (95% CI: 0.30–0.83) with a cutoff value of 3.1 g/dL; for lymphocyte count, it was 0.61 (95% CI: 0.57–0.84) with a cutoff value of 0.34 × 10^3^/µL; and for PNI, it was 0.58 (95% CI: 41.18–85.06) with a cutoff value of 34.6.

**Conclusion:**

The low PNI group exhibited a significantly higher mortality rate; nonetheless, PNI alone does not hold clinical significance as a prognostic marker. However, when combined with other clinical parameters, it can contribute to a more comprehensive assessment of patients.

## Background

Heart failure, a critical cardiovascular condition, poses a significant threat to both the Turkish and global populations, necessitating comprehensive treatment approaches and contributing to elevated mortality rates. It refers to the sudden onset or worsening of heart failure symptoms, such as shortness of breath, fluid retention, and fatigue, often requiring immediate medical attention. It can occur as a result of a sudden cardiac event, such as myocardial infarction, or the acute decompensation of chronic heart failure. The characterization of heart failure as the ultimate stage of severe cardiac ailments underscores its clinical importance [1, 2]. Epidemiological studies have reported varying incidences of heart failure between genders, with some indicating higher rates in women while others suggest a predilection for men [3–5]. In the United States, healthcare costs associated with hospitalizations of individuals aged 65 and above constitute a substantial portion, amounting to 80% of the total expenses related to heart failure [6].

Anemia and renal dysfunction are recognized factors influencing the prognosis of heart failure, further complicating its management [6, 7]. Additionally, emerging evidence from several studies underscores the independent prognostic impact of malnutrition on heart failure outcomes [8, 9].

The prognostic nutritional index (PNI) emerged in 1980 as a metric rooted in malnutrition assessment [10]. Onodera et al. proposed a simplified calculation method for PNI using albumin and lymphocyte values, initially introducing it as an indicator for postoperative complications [11]. While initially applied in gastrointestinal surgery, subsequent investigations explored its potential as a prognostic marker in diverse medical fields, including colorectal cancer surgery [12], renal cell cancer surgery [13], chronic renal failure [14], orthopedic patients with fractures [15], sepsis patients [16], ischemic stroke patients [17], and even in individuals diagnosed with COVID-19 [18, 19]. Lower PNI values indicate poor nutritional status and have been associated with worse outcomes.

In the realm of cardiovascular diseases, studies have deliberated on the prognostic significance of PNI scores in patients with acute coronary syndrome [20] and, notably, in those diagnosed with heart failure, a condition garnering increased attention in recent years [9, 21, 22].

Geriatric typically refers to individuals who are aged 65 and older. This age threshold is widely used in clinical practice and research to categorize the older population. However, geriatric care focuses not only on age but also on the overall health status and functionality of the individual, recognizing that chronological age may not fully capture the complexities of aging. Nutrition plays a crucial role in geriatric care, as malnutrition or poor dietary habits can significantly impact an older person’s health, contributing to muscle loss, frailty, and increased risk of illness. Maintaining adequate nutrition is essential for preserving functionality, preventing disease, and improving quality of life in the elderly, underscoring the importance of individualized dietary assessments in geriatric care [23–25]. This study aims to assess the impact of PNI on the prognosis of geriatric patients diagnosed with acute heart failure.

## Methods

### Study design

This single center, retrospective study focused on geriatric patients presenting to the emergency department between January 1, 2022, and December 31, 2022, exhibiting clinical, radiological, and laboratory manifestations of acute heart failure.

### Study population

The study population included patients aged 65 and above, and data included clinical assessments, radiological findings, and laboratory results, particularly hemogram and biochemical parameters such as lymphocyte count and albumin levels, documented in the emergency department. The study simple consisted of 104 patients, 60 female and 44 males.

Patients presenting to the emergency department during the year 2022 with clinical and radiological evidence of acute heart failure (Class III and class IV New York Heart Association (NYHA)) patients were included.

NYHA Classification—The stages of heart failure:

Class I—No symptoms and no limitation in ordinary physical activity, e.g., shortness of breath when walking, climbing stairs, etc.

Class II—Mild symptoms (mild shortness of breath and/or angina) and slight limitation during ordinary activity.

Class III—Marked limitation in activity due to symptoms, even during less-than-ordinary activity, e.g., walking short distances (20—100 m). Comfortable only at rest.

Class IV- Severe limitations. Experiences symptoms even while at rest. Mostly bedbound patients [26].

Diuretics, ACE (angiotensin-converting enzyme) inhibitors and noninvasive mechanical ventilation were used in the treatment of all patients.

Exclusion criteria encompassed individuals with additional diagnoses other than acute heart failure, patients who developed heart failure while being followed in the emergency department due to drug reaction, allergic shock reaction and sepsis. Other exclusion criteria and summary of exclusion criteria are presented in Table 1.

**Table 1:** Exclusion criteria

Exclusion criteria	Explanation
Patients with comorbid diseases	Patients with serious comorbidities such as renal failure, malignancy, or liver cirrhosis were excluded from the study
Heart failure developed due to drug reaction or allergic shock	Cases where heart failure developed due to drug side effects or allergic reactions were not included in the study
Patients with heart failure caused by trauma	Heart failure cases resulting from trauma were excluded from the study
Patients with incomplete data	Patients with missing or incomplete medical records or insufficient clinical data were excluded from the study
Patients who refused treatment	Patients who refused to participate in the treatment process or did not comply with clinical treatment protocols were excluded from the study
Patients referred to another clinic from the emergency department	Patients with acute heart failure who were referred to another clinic from the emergency department were excluded due to different treatment protocols
Patients with unavailable mortality data	Patients whose hospital mortality data could not be accessed were excluded from the study
Patients diagnosed with sepsis	Patients diagnosed with sepsis alongside acute heart failure were excluded from the study

### Data collection

Patient data encompassed age (years), gender, comorbidities, white blood cell count, neutrophil and lymphocyte counts, hemoglobin, hematocrit, neutrophil–lymphocyte ratio, sodium, potassium, albumin, C-reactive protein, brain natriuretic peptide levels, and the PNI. PNI scores were calculated using the formula: PNI = 10 × serum albumin (g/dl) + 0.005 × total lymphocyte count (/mm^3^). Additionally, 30-day mortality rates were recorded, and patients were categorized into survivor and non-survivor groups based on data obtained from the National Death Notification System in Turkey.

### Statistical analysis

Categorical data were analyzed using Chi-square and Fisher’s exact tests, while quantitative variables were presented as number and percentile. Continuous data presented as median and interquartile range (IQR, 25–75th percentile) values. The Mann–Whitney test was employed to analyze paired groups. The correlation between the low PNI and 30-day mortality was assessed using Spearman’s rank correlation coefficient (Spearman’s rho). Area under the curve (AUC) values were calculated during the receiver operating characteristic (ROC) analysis, and cutoff, sensitivity, specificity, accuracy, and 95% confidence interval (CI) data were assessed. Patients were grouped as low PNI and high PNI groups, according to the cutoff point (35.6) calculated by ROC analysis. Youden’s index was used to determine the optimal cutoff value for scores with highest sensitivity and specificity. Likelihood ratios were calculated using sensitivity and specificity values in the evaluation of relationship between 30-day mortality and scoring systems. Statistical analyses were conducted using the jamovi (Version 2.5) [Computer Software] Retrieved from https://www.jamovi.org.), with significance at *p* < 0.05. The odds ratio (OR) and its 95% confidence interval (CI) were calculated following the methodology outlined by Altman (1991) [27].

## Results

A total of 104 patients were included in the study. The 30-day mortality rate was 19.24%. The overall median age for the entire cohort was 78.5 years (IQR: 71.8—85). The median age of the patients in the high PNI group (≥ 35.6) was 77 years (IQR: 71—84.5), while the median age in the low PNI group (< 35.6) was 82 years (IQR: 75—87). There was no statistically significant difference in age between the two groups (*p* = 0.103).

For the total cohort, 60 patients (57.7%) were females, and 44 patients (42.3%) were males. In the high PNI group, 47 patients (59.5%) were females, and 32 patients (40.5%) were males. In the low PNI group, 13 patients (52%) were females, and 12 patients (48%) were males. The comparison between gender distribution in the two groups did not show a statistically significant difference (*p* = 0.668).

The median ejection fraction value in the survivor group was 50%, whereas in the non-survivor group, it was 40%. Despite this difference, no statistically significant difference was found between low PNI and high PNI groups.

A statistically significant difference was identified between high and low PNI groups in terms of lymphocyte count, neutrophil–lymphocyte ratio, C-reactive protein, and albumin (*p* values: < 0.001, < 0.001, 0.011, and < 0.001, respectively, Mann–Whitney U test).

The 30-day mortality of geriatric acute heart failure patients in the low PNI (< 35.6) group was higher than those in the high PNI (≥ 35.6) group (*p* value: 0.032).

Comparison of demographic, comorbidity, laboratory parameters, and mortality in geriatric patients with acute heart failure between high and low PNI groups is presented in Table 2.

**Table 2:** Comparison of demographic, comorbidity, laboratory parameters, and mortality in geriatric patients with acute heart failure between high and low PNI groups

	High PNI ≥ 35,6 (*n* = 79, 76%)	Low PNI < 35,6 (*n* = 25, 24%)	Total (*n* = 104)	*p* value
Age, median (25–75th percentile)	77 (71–84.5)	82 (75–87)	78.5 (71.8–85)	0.103*
*Gender* (n, %)				
Female	47 (59.5%)	13 (52%)	60 (57.7%)	0.668**
Male	32 (40.5%)	12 (48%)	44 (42.3%)	
*Comorbidities* (n, %)				
Chronic obstructive pulmonary disease	18 (22.8%)	5 (20%)	23 (22.1%)	1.000**
Hypertension	39 (49.4%)	13 (52%)	52 (50%)	0.455**
Diabetes mellitus	35 (44.3%)	7 (28%)	42 (40.4%)	0.070**
Ischemic heart disease	20 (25.3%)	3 (12%)	23 (22.1%)	0.080**
Congestive heart failure	49 (62%)	12 (48%)	61 (58.7%)	0.534**
Chronic renal failure	18 (22.8%)	5 (20%)	23 (22.1%)	1.000**
*Laboratory parameters, median (25–75th percentile)*				
Ejection fraction (%)	50 (35–55)	42.5 (30–50)	45 (31.2–55)	0.235*
White blood cell count (10^3^ µ/L)	9.1 (7–12.6)	9.1 (7.4–11.1)	9.1 (7–12.2)	0.773*
Neutrophil count (10^3^ µ/L)	6.5 (4.9–9.7)	7.5 (5.8–9.9)	6.6 (5–9.9)	0.285*
Lymphocyte count (10^3^ µ/L)	1.5 (1–2.3)	0.8 (0.3–1.2)	1.3 (0.8–1.8)	< 0.001*
Hematocrit (%)	36 (31.8–39.3)	33.8 (28.6–39)	35.5 (31.2–39.3)	0.126*
Hemoglobin (g/dl)	11.1 (10.1–12.5)	9.9 (9.2–12.1)	11.1 (9.8–12.4)	0.095*
Neutrophil–lymphocyte ratio	4.4 (2.7–7)	11.1 (7.4–19.9)	5.2 (3.2–8.7)	< 0.001*
C-reactive protein (mg/L)	1.4 (0.6–5)	6.5 (1.2–11.1)	1.8 (0.6–6.5)	0.011*
Sodium (mEq/L)	138 (135–140.5)	138 (132–141)	138 (135–141)	0.362*
Potassium (mEq/L)	4.9 (4.5–5.4)	4.9 (4.4–5.4)	4.9 (4.5–5.4)	0.879*
Brain natriuretic peptide	1068.6 (706.5–1851)	1612.2 (787.3–1923.5)	1158.8 (728.7–1876.8)	0.237*
Albumin (g/dl)	3.5 (3.3–3.9)	2.7 (2.6–3)	3.4 (3.2–3.8)	< 0.001*
*Mortality* (n, %)	11 (13.9)	9 (36)	20 (19.2)	0.032**

Values in bold are considered statistically significant

*Mann–Whitney *U* test

**Chi-square test

**Fig. 1:**
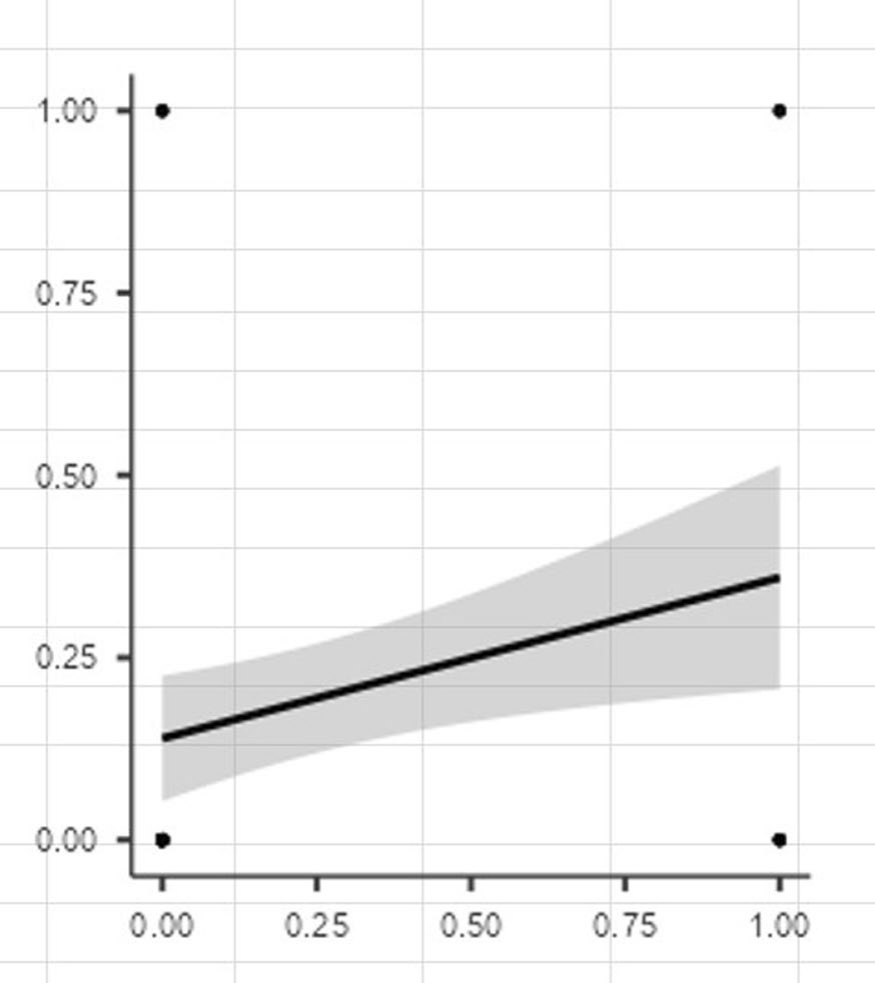
The correlation matrix of PNI and mortality

The correlation matrix in the provided Fig. 1 indicates the relationship between the low PNI and mortality, analyzed using Spearman’s rank correlation coefficient. A positive correlation of 0.239 was observed between the low PNI values and mortality (*p* value: 0.014, Spearman’s correlation test).

The odds ratio for PNI (< 35.6) was calculated as 3.48, with a 95% confidence interval of 1.23–9.80 (*p* = 0.015).

Given the statistical significance of lymphocyte count, albumin, and PNI in relation to mortality, ROC analysis was performed. The results from the diagnostic test performance analysis indicated that these variables were statistically significant predictors of mortality. The AUC values were calculated as 0.53 (95% CI: 0.30–0.83) for albumin, with a cutoff value of 3.1 g/dL; 0.61 (95% CI: 0.57–0.84) for lymphocyte count, with a cutoff value of 0.34 × 10^3^ µ/L; and 0.58 (95% CI: 41.18–85.06) for PNI, with a cutoff value of 34.6 (Table 3).

**Table 3:** ROC analysis and diagnostic accuracy studies for predicting 30-day mortality

	Lymphocyte count			Albumin			PNI		
Decision Statistics	Estimate	Lower	Upper	Estimate	Lower	Upper	Estimate	Lower	Upper
Apparent prevalence	6.7	2.7	13.4	19.2	12.2	28.1	16.3	9.8	24.9
True prevalence	19.2	12.2	28.1	30	12.2	28.1	19.2	12.2	28.1
Sensitivity	20	5.7	43.7	83.3	11.9	54.3	35.0	15.4	59.2
Specificity	96.4	89.9	99.3	73.1	73.6	90.6	88.1	79.2	94.1
Diagnostic accuracy	81.7	72.9	88.6	30	63.5	81.3	77.9	68.7	85.4
Positive predictive value	57.1	18.4	90.1	83.3	11.9	54.3	41.2	18.4	67.1
Negative predictive value	83.5	74.6	90.3	16.7	73.6	90.6	85.1	75.8	91.8
Proportion of false positives	3.6	0.7	10.1	70	9.4	26.4	11.9	5.9	20.8
Proportion of false negative	80	56.3	94.3	70	45.7	88.1	65.0	40.8	84.6
False Discovery Rate	42.9	9.9	81.6	16.7	45.7	88.1	58.8	32.9	81.6
False Omission Rate	16.5	9.7	25.4	19.2	9.4	26.4	14.9	8.2	24.2
Diagnostic odds ratio	6.750	13.764	33.103	2.143	0.702	6.537	3.985	12.852	12.354
Number needed to diagnose	6.087	−229.841	2.330	7.500	−6.903	2.229	4.330	−184.687	1.874
Youden's index	0.164	−0.0435	0.429	0.133	−0.145	0.449	0.231	−0.0541	0.534
Likelihood ratio of a positive test	5.600	13.600	23.059	1.800	0.791	4.098	2.940	12.772	6.768
Likelihood ratio of a negative test	0.830	0.6638	1.037	0.840	0.621	1.137	0.738	0.5299	1.027

## Discussion

In this study, low PNI demonstrated prognostic value in geriatric patients presenting with acute heart failure, as it was significantly associated with mortality. However, the AUC values were not sufficient to establish PNI as an independent prognostic marker.

In our analysis, we initially employed nonparametric comparison tests to assess the relationship between low PNI and mortality. The low PNI group exhibited a significantly higher mortality rate compared to the high PNI group. Subsequently, we conducted further analysis using the ROC curve to evaluate the PNI’s ability to differentiate between patients who survived and those who did not. AUC values less than 0.5 were deemed indistinguishable from random, while values approaching 1 indicated a near-perfect model. Literature suggests that an AUC value greater than 0.8 is necessary for a model to predict mortality effectively [28]. In our discriminatory power analysis, we determined the AUC value for PNI to be 0.58, which is considered unacceptable.

Likelihood ratios provide the clearest data on the reliability of scoring systems. Likelihood ratios above 5 and below 0.2 are regarded as strong indicators to rule in or rule out diagnoses, respectively [29]. In our study, we found likelihood ratios values of 2.9 and 0.73, indicating that PNI cannot be used in isolation for clinical decision-making. Therefore, our retrospective comparative study demonstrates that while the low PNI group showed a significantly higher mortality rate, it was not clinically significant.

The initial use of PNI by Onodera et al. in assessing the operation risk in malnourished patients receiving preoperative total parenteral nutrition demonstrated its potential utility, suggesting contraindication for surgery if PNI was below 40 [11]. Subsequent studies explored PNI in different medical contexts, such as colorectal cancer, renal cell cancer, chronic renal failure, femoral fracture surgery, sepsis, ischemic stroke, and even in the context of COVID-19. These investigations often revealed significant correlations between PNI and outcomes, including mortality [12–18].

Ellez et al. estimated the effect of the PNI on the prognosis of metastatic castration-sensitive prostate cancer. Their univariate test results revealed a relationship between PNI and overall survival, with a hazard ratio of 1.8, consistent with the findings of the current study [30]. Similarly, Lu et al. examined the relationship between PNI and all-cause mortality in critically ill patients. Their univariate test results also demonstrated a connection between PNI and overall survival; however, the AUC was 0.6, aligning with the results of the present study [31].

Recent research has indicated increased studies focusing on the relationship between heart failure and PNI in the last two years [21]. A meta-analysis involving heart failure patients suggested that low PNI was significantly associated with increased mortality, emphasizing its potential role as a biomarker for determining mortality risk. Additionally, associations between low albumin and calcium levels in malnutrition affecting immunity were highlighted [21].

In a prospective study with heart failure patients, age, gender, sodium, systolic blood pressure, and PNI were identified as independent indicators of cardiovascular mortality [22]. Another study by Kawata et al. found that higher PNI and albumin levels at discharge in heart failure patients were associated with a better prognosis, emphasizing the potential use of these parameters as prognostic indicators [9]. Conversely, our study observed a significant relationship between PNI, albumin, lymphocyte levels, and mortality.

It is crucial to acknowledge the limitations of our retrospective design, which prevented the evaluation of changes in laboratory parameters over time and the consideration of additional diseases affecting these parameters in the geriatric population. PNI could change during hospitalization. A second limitation is we did not take note of changes on PNI. Our results could not generalize to changes of PNI or PNI measured during hospitalization.

## Conclusions

Our results indicate that while the low PNI (< 35.6) group showed a significantly higher mortality rate, PNI alone lacks sufficient clinical significance as a standalone prognostic marker. However, when combined with other clinical parameters, it can contribute to a more comprehensive assessment of patients. Additionally, caution should be exercised in generalizing these findings. Further research, ideally with a prospective design and larger sample size, is required to validate our results.

## Data Availability

All data generated or analyzed during this study are included in this published article.

## Abbreviations

ACE: Angiotensin-converting enzyme
AUC: Area under the curve
CI: Confidence interval
IQR: Interquartile range
NYHA: New York Heart Association
OR: Odds ratio
PNI: Prognostic nutritional index
ROC: Receiver operating characteristics
